# Research on a Variable Pressure Driving Method for Soft Robots Based on the Electromagnetic Effect

**DOI:** 10.3390/s23146341

**Published:** 2023-07-12

**Authors:** Zhongyuan Zhang, Lei Zhang, Mingjing Guan, Shuai Zhang, Tengfei Jiao

**Affiliations:** Department of Automation, College of Engineering, Ocean University of China, Qingdao 266404, China; zzy8203@stu.ouc.edu.cn (Z.Z.); gmj8094@stu.ouc.edu.cn (M.G.); zs6419@stu.ouc.edu.cn (S.Z.); jiaotengfei@stu.ouc.edu.cn (T.J.)

**Keywords:** pneumatic drive, electromagnetic effect, connected structure, soft robotics

## Abstract

This study proposes a novel variable air pressure supply structure based on the electromagnetic effect. This structure can be implemented in various soft robots driven by air pressure, including pneumatic artificial muscles, pneumatic soft grippers, and other soft robots. The structure’s main body comprises a hollow circular tube, a magnetic piston arranged in the tube, and an electromagnetic solenoid nested outside the tube. The electromagnetic solenoid is designed with special winding and power supply access modes, generating either an attractive force or a repulsive force on the magnetic piston. This solenoid conforms with the magnetic piston expectation in the tube by changing the polarity direction. The interior of the whole structure is a closed space. The gas is conveyed to the soft robot by the gas guide hoses at the two ends of the structure, and the expansion energy of the compressed gas is fully utilized. Then, the gas supply pressure is controlled to drive the robot. The mathematical model of the structure is established based on the analysis of the electromagnetic force and gas pressure on the piston. The simulation results show that the structure’s inherent vibration characteristics under various parameters align with expectations. The real-time automatic optimization of the controller parameters is realized by optimizing the incremental proportional-integral-derivative (PID) controller based on a neural network. The simulation results show that the structure can meet the application requirements. The experimental results show that the proposed gas supply structure can provide a continuous pressure supply curve with any frequency in a specific amplitude range and has an excellent tracking effect on the sinusoidal-like pressure curve.

## 1. Introduction

Soft robotics has become increasingly popular due to its advantages, such as high flexibility, strong adaptability, and high safety [1,2]. This technology is widely used in medical rehabilitation [3,4,5], adaptive grasping [6,7,8], deep-sea exploration [9], and com-plex environment exploration [10]. However, driving soft material that is easy to deform and difficult to control is an important research topic. Researchers have explored various options such as electroactive polymer (EAP) [11], ionic polymer metal composite (IPMC) actuators, shape memory alloy (SMA) actuation, and soft robots based on chemical exergy reactions. Each approach has its strengths and limitations. For example, EAP has fast response speed and excellent bionic performance but is greatly affected by temperature as it cannot withstand high temperatures [12,13,14,15]. IPMC actuators for soft underwater robots [16,17] have the characteristics of large deformation and low driving voltage. However, its energy density is small, and the response speed is slow [18,19]. SMA actuation uses materials corresponding to different deformations at different temperatures to generate the driving force of soft robots. SMA actuation is sensitive to temperature and has strong environmental adaptability, but the problem of limited driving force must be solved [20,21]. The soft robot based on chemical exergy reaction has a high reaction rate and can realize fast driving, but it is difficult to control its movement accuracy [22,23,24].

The pneumatic drive principle involves using changes in gas pressure to move the robot by deforming the soft material [25]. The pneumatic drive is the most common and effective drive mode due to easy gas availability, its lightweight nature, and its non-polluting characteristic [26]. In order to realize the application of pneumatic soft robots in different environments, researchers have made a series of explorations focusing on materials, structures and controls. Pneumatic artificial muscle (PAM) designed by McKibben is used as a rehabilitation and orthodontic device for patients [27]. Guan et al. drew inspiration from the arrangement of elephant trunk muscle fibers to study extensional and contractile PAM actuators. The authors used this inspiration to create a series of extensional and contractile bending or spiral PAMs by introducing additional constraint structures [28]. Ge et al. proposed the design of soft fabric-based pneumatic actuators (SFPAs) to study the mechanical characteristics of various knitted and woven fabric structures and design three structures: thumb abduction, finger bending, and finger extension. SFPAs were integrated into a flexible wearable auxiliary glove with a portable control system [29]. Inspired by octopus tentacles, Xie et al. proposed a conical elastic air chamber software driver and combined this with vacuum suction cups to flexibly grasp objects of various shapes and materials [30].

Wang et al. recently advanced the study of negative pressure-driven soft spherical grippers, using the principle of particle blockage. These authors proposed a new grasping strategy that combined positive pressure inflation and negative pressure grasping to enhance the grasping performance without modifying the gripper. The feasibility of this approach was confirmed through experimental tests and theoretical analysis [31]. Yap et al. used commercial open-source, fused deposition modeling technology to print the whole pneumatic software driver simultaneously using a flexible wire with a Shore hardness of 85a. Extending the corrugated structure on one side of the air cavity under internal pressure, the driver can bend to one side [32]. Lee and Rodrigue proposed a novel origami-based vacuum pneumatic artificial muscle using a metal frame as the endoskeleton. Their actuator is made of a sealed origami membrane cavity. Their membrane cavity is supported by a uniformly spaced transverse metal frame internally. Under vacuum, the tension on the wall surface of the actuator and negative vertical pressure on the base plate of the actuator can provide a larger contraction force with a maximum contraction ratio of 95%. A contraction ratio of 87% can be obtained at a load of 400 N at 60 kPa [33].

To sum up, although the soft robot has made many research achievements and breakthroughs, in some aspects, especially the reliability of the pneumatic soft robot system, gas supply and motion control problems restrict the practical application of the pneumatic soft robot.

1. A soft body drive structure generates air pressure through self-deformation so as to achieve that purpose of applying work to the outside, so the special structure and manufacturing material of the soft body drive structure needs to be subjected to repeat large deformation, and the problems of fatigue damage and durability reduction of the traditional soft body driver are caused.
2. Pneumatic soft robots mostly use air compressors to supply pressure directly, which are large in size and high in energy consumption. For pneumatic soft robots with strong mobility, this pressure supply mode greatly limits their application scenarios and working performance. It is difficult to apply it to ruins search, underwater exploration and other scenes, and it is difficult to achieve the transformation from indoor to outdoor.
3. In the control algorithm, we can establish a certain control model through the study of the nonlinear problem of the relatively simple soft robot system, but for the more complex soft robot system, it often faces enormous difficulties. At the same time, because of the special structure, easy deformation of the material, strong coupling and nonlinearity, the traditional control method has low control accuracy, cannot meet the application requirements, and the control model is difficult to establish.

Li et al. [34] proposed a connected design scheme of gas supply structure based on an electromagnetic effect drive in our laboratory. Creating a reciprocating air pressure supply characteristic could enhance the PAM-driven legged robot’s rhythmic motion.

This study presents an evolutionary pneumatic supply structure based on the electromagnetic effect, designed explicitly for PAM, soft gripper, and soft pneumatic robots, with the following characteristics:

1. Light structure, small volume and convenient assembly. Compared with the traditional pressure supply method, the pressure supply structure proposed in this paper does not need to be equipped with other complex devices, and can be more convenient to carry on outdoor equipment.
2. The energy stored by the compressed gas can be fully utilized to reduce the comprehensive energy consumption of the system. In the process of movement, the energy stored when the gas is compressed will push the piston back and forth. This phenomenon reduces the input of external energy in the subsequent movement.
3. The supply pressure curve has continuity. In practical application, in order to ensure the stability and less vibration of the engine block, the output pressure of the pressure supply structure is basically required to be limited, continuous and without obvious step. The air pressure curve generated by the structure is realized through the movement of the piston; the mode ensures the continuity of the pressure supply curve in principle, and the pressure curve is not required to be limited to jump in other modes.
4. The neural network PID controller based on input and feedback can adjust the parameters adaptively, and improve the robustness and reliability of the system. The introduction of piston position and its motion state can produce good prediction ability for model parameters and controller output.

The remainder of this paper is organized as follows: Section 2 analyzed the working principles of the gas supply structure, focusing on the design of the electromagnetic and piston structures. Section 3 establishes a mathematical model of the gas supply structure based on an analysis of the forces on the piston. Section 4 presents the physical design of the gas supply model and describes the design methods for the electromagnetic and piston structures. Section 5 analyzes the effects of model parameters on the inherent characteristics of the structure, proposes a control method, and conducts pneumatic control simulations. Section 6 presents a physical platform to verify the practical applicability of the structure. Finally, the conclusion summarizes the contents of the article.

## 2. Structure Design

### 2.1. Overall Mechanical Design

**Figure 1:**
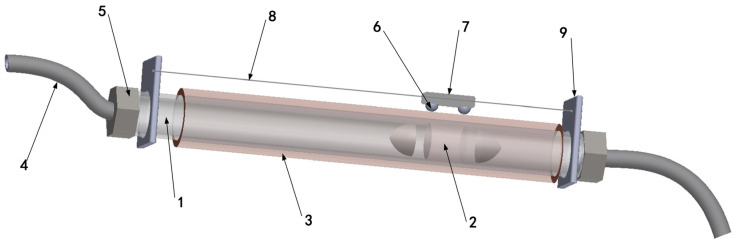
Overall gas supply structure: 1. Cylindrical Tuber. 2. Magnetic Piston. 3. Electromagnetic Solenoid. 4. Air Guide Hoses. 5. Connecting Cap. 6. Magnetic Globules. 7. Fixed Cap. 8. Track Wire. 9. Guideway Fixing Plate.

The gas supply structure comprises an inner smooth-walled cylindrical tube, an internal magnetic piston, and an outer embedded electromagnetic coil, forming a sealed space inside the overall structure, as shown in Figure 1. The cylindrical tube (1) has air guide hoses (4) on both ends for transporting gas to the soft robot structure. At both ends of the magnetic piston, the change in gas volume (2) affects the gas pressure on the left and right sides, generating different pressures for driving the piston back and forth. By adjusting the input current, the electromagnetic solenoid (3) embedded in the outer wall of the cylindrical tuber can produce different attractive or repulsive forces on the magnetic piston, ultimately affecting the motion of the piston inside the tube and achieving the purpose of adjusting the gas supply pressure. The outer magnetic ball (6) adheres to the coil’s outer surface through the magnetic piston’s force of attraction (2). Moreover, this system changes its contact position with the motion of the internal piston. The two magnetic globules serve as the connecting points for the two poles of the coil power supply, and their positions change to energize part of the coil changes accordingly, allowing the coil to continue exerting control force on the magnetic piston. The fixed cap (7) outside the magnetic ball is used to keep the distance between the two magnetic globules constant. The tracking wire (8) passes through the magnetic ball fixed cap, fixes on the circular tube fixed plates at both ends, and forms a trajectory line.

The piston divides the tube into two closed chambers. The changing force applied to the piston affects its movement state, outputting its required air pressure curve. When the piston moves to the right, the gas in the right chamber is compressed, resulting in a higher pressure than in the left chamber. Then, the pressure difference pushes the piston to the left. Even without external input, the piston can move for several cycles, producing reciprocating air pressure output in the unbalanced state.

### 2.2. Analysis and Design of Electromagnetic Structure

The electromagnetic solenoid is nested outside the circular tube and has the following characteristics:

1. The magnetic field intensity of the solenoid is directly proportional to the current, and the direction of the magnetic field conforms to the Ampere rule.
2. When the current is constant, the magnetic field strength decreases significantly with the increase in distance.
3. When the solenoid is energized, the magnetic field intensity inside the structure reaches the maximum level and is uniformly distributed.

This is crucial to allow the piston to move continuously within the tube without fluctuation in the force applied. Additionally, sufficient electromagnetic force must be applied to the magnetic piston to produce higher air pressure. These design objectives have been carefully considered and implemented to ensure optimal performance and reliability of the electromagnetic structure, as discussed below:

- The position of the solenoid to which the power is applied can be changed with the movement of the piston.
- When the control voltage is small, the solenoid can generate enough electromagnetic force for the magnetic piston.

**Figure 2:**
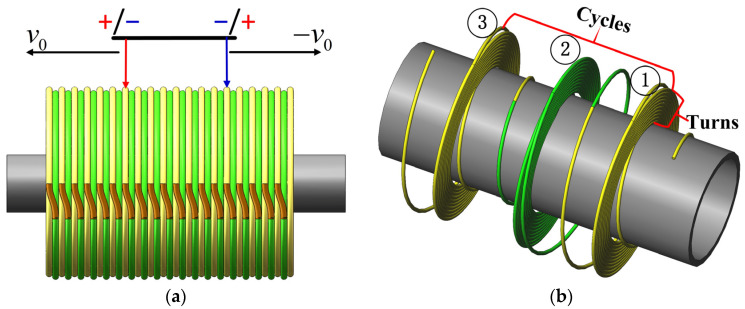
Electromagnetic structure design: (**a**) Access point of the power supply is located at the outermost side of the solenoid, moving with the movement of the internal magnet. (**b**) Solenoid model adopts the winding mode of the first number of turns and the number of cycles.

A sliding power supply access mode is adopted for the solenoid to make the solenoid exert a continuous force on the magnetic piston, as shown in Figure 2a. The coil power access point is positioned outside the solenoid and moves along with the movement of the magnet. The external power access point also moves when the magnet moves under the action of the solenoid magnetic field, which is favorable for controlling the long stroke and durability of the magnet. However, the conventional winding mode only allows for one turn of the outermost layer, requiring a larger control voltage to produce enough control force on the magnetic piston, which can compromise the equipment’s safety. To cooperate with the sliding connection mode, a new solenoid winding model is designed to generate enough electromagnetic force under the premise of a smaller control voltage, as shown in Figure 2b.

The solenoid model adopts a winding mode of the first winding number. Then, n turns from the inner layer to the outer layer and wounds the winding number of the first winding of the copper wire. The second winding is wound along the axial direction. Moreover, the first and second windings are connected to the outermost layer. The second and third windings are connected to the innermost layer. When this winding mode is combined with the sliding connection mode, the effective access turns of the solenoid can be n turns, solving the problem of insufficient electromagnetic force generated by the single-turn coil.

**Figure 3:**
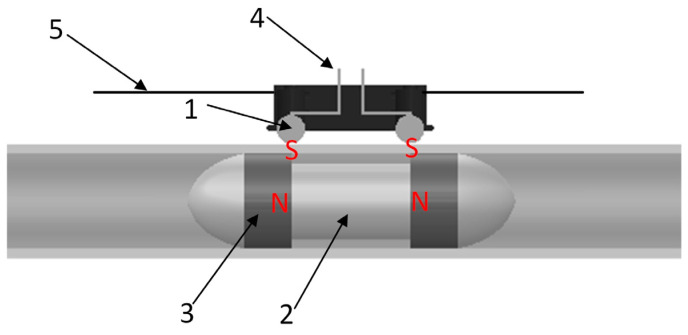
Electromagnetic structure model: 1. Magnetic Globule. 2. Middle Support. 3. Cylindrical Magnet. 4. Power Leads. 5. Guide Rail.

Figure 3 shows the electromagnetic structure designed in this paper.

The magnetic piston consists of a cylindrical permanent magnet with two opposite magnetic poles and an adiabatic magnetic balance weight. However, the piston’s overall length can be greater than that of the energized portion of the solenoid by adjusting the length of an intermediate magnetically insulating weight. Two spherical permanent magnets are arranged outside the solenoid, and the fixing cap is clamped to maintain the same distance between the two magnetic balls, keeping the electrified part of the solenoid unchanged. The magnetic ball has excellent performance and is securely attached to the outer wall of the solenoid under the internal magnet attraction. Upon connecting the power supply, the internal magnetic piston moves, and the magnetic ball follows suit under the piston and the internal magnet attraction, changing an access point of a solenoid power supply.

**Figure 4:**
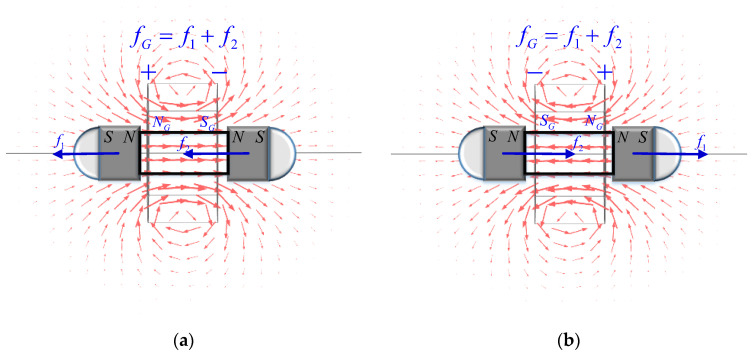
Electromagnetic structure motion characteristic: (**a**) When the piston is energized in the positive direction, the total external force on the piston is directed to the left. (**b**) The total external force exerted on the piston when energized in the reverse direction is to the right.

Figure 4 shows the magnetic field distribution generated by the electromagnetic structure and the movement effect of the piston.

When the solenoid is energized in the forward direction, as shown in Figure 4a, the left side of the energized part is the N pole, and the right side is the S pole, generating a leftward repulsive force $f_{1}$ on the permanent magnet on the piston’s left side. At the same time, an attractive leftward force $f_{2}$ is generated on the permanent magnet on the piston’s right side. In this state, the energized solenoid generates the total electromagnetic combined external force. The entire piston is $f_{G}=f_{1}+f_{2}$, pushing the entire piston to the left to move. Figure 4b shows that reversing the magnetic field changes its direction. This causes the left side of the solenoid to become the S pole and the right side to become the N pole. This process generates a rightward electromagnetic force that moves the piston to the right. By changing the direction of the current, the piston can be controlled to move to the left and right within the sealed chamber. This scenario generates air pressure that changes reciprocally in a certain range, allowing the air pressure supply to be achieved.

## 3. Modeling

Assuming that the displacement of the piston at the middle position of the tube is x=0, the movement to the right side is positive, and the movement to the other side is negative. When the piston is at x, the gas volume at both ends of the tube is as follows:

(1) $$\begin{array}{l} V_{l}=V_{l0}+sx\\ V_{r}=V_{r0}-sx \end{array},$$

where $V_{l}$ and $V_{r}$ are the volumes of the tube at the left and right ends of the piston, respectively. $V_{l0}$ and $V_{r0}$ are the volumes of the left and right containers, respectively, when the piston is positioned at the center of the tube. Moreover, $V_{l0}\approx V_{r0}$; s is the area of the piston, and x is the displacement of the piston, based on the state equation of ideal gas:

(2) $$pV=nrT_{emp},$$

where p is the gas pressure in Pa. V is the volume occupied by the gas in $m^{3}$; n is the mass of the gas, in mol. $T_{emp}$ is the system temperature in K; and r is the gas constant (constant of proportionality) in J/(mol⋅K). For a gas mixture, such as air, the pressure p is a linear combination of the partial pressures of its components. Thus, (2) is rewritten as follows:

(3) $$pV_{\mathrm{air}}=n_{\mathrm{air}}rT_{emp}.$$

By combining (2) and (3), we develop the following:

(4) $$\begin{array}{l} p_{l}=\frac{n_{\mathrm{airl}}rT_{emp}}{V_{l0}+sx}=\frac{k_{l}(T_{emp})}{V_{l0}+sx}\\ p_{r}=\frac{n_{\mathrm{airr}}rT_{emp}}{V_{r0}-sx}=\frac{k_{r}(T_{emp})}{V_{r0}-sx} \end{array},$$

where $k_{l}(T)=n_{air1}rT_{emp}$, $n_{air1}$ is the amount of air substance at the left end of the container. This amount is constant and does not change after the container is sealed and the displacement of air occurs. The same applies to the right end. Thus, $p_{l}$ and $p_{r}$ are the gas pressures at the left and right ends of the piston, respectively, which function with the displacement x and the ambient temperature $T_{emp}$. In the same use scenario, the temperature of the muscle working environment does not change dramatically. In addition, the thermodynamic temperature base is large, and the temperature change range in the general application is relatively small, which is constant in simulation.

(5) F=ps

The relationship between the pressure and the mixed gas pressure is given in (5). Substituting (4) with (5) gives the following:

(6) $$\begin{array}{l} f_{l}=\frac{k_{l}(T_{emp})}{V_{l0}+sx}s\\ f_{r}=\frac{k_{r}(T_{emp})}{V_{r0}-sx}s \end{array},$$

where $f_{l}$ and $f_{r}$ are the forces exerted on the piston by the air pressure at the piston’s left and right ends, respectively. The resultant force is the gas pressure applied to the piston at displacement x, given by

(7) $$f=f_{r}-f_{l}.$$

**Figure 5:**
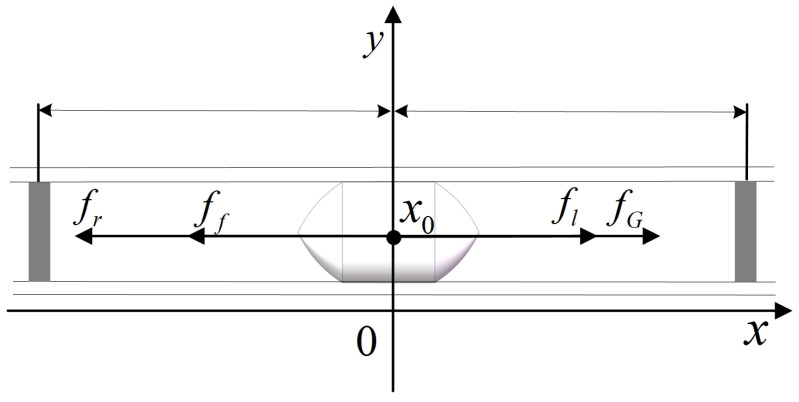
Piston force analysis diagram.

In the horizontal direction of the tube, the piston is subjected to four forces: the pressure $f_{l}$ and $f_{r}$ exerted by the gas on both sides, the dynamic friction force $f_{f}$, and the magnetic attraction force $f_{G}$. Then, $x_{0}$ is the initial piston position. Figure 5 shows the force analysis of the piston.

(8) $$\left\{\begin{array}{l} f-f_{f}-f_{G}=ma\\ \ddot{x}=a\\ x_{t=0}=x_{0}\\ {\dot{x}}_{t=0}=0 \end{array}\right.$$

Equations (6) and (7) are substituted with (8) to obtain

(9) $$x=x_{0}+\frac{1}{m}\int_{0}^{t}\int_{0}^{t}(\frac{k_{r}(T_{emp})}{V_{r0}-sx}s-\frac{k_{l}(T_{emp})}{V_{l0}+sx}s-f_{f}-f_{G})dtdt.$$

The dynamic friction force must be measured in the application as it is related to the material and manufacturing process. Moreover, the electromagnetic structure is nested outside the tube and is used to control the piston’s movement position in the tube. By applying the Biot-Savart law [35], we can accurately determine the magnetic induction at any point of the ring current, expressed as follows:

(10) $$B_{x}=\frac{\mu_{0}}{2}\frac{R^{2}I}{{\left(R^{2}+l^{2}\right)}^{\frac{3}{2}}},$$

where $\mu_{0}$ is vacuum permeability, whose value is $4\pi \times 10^{-7}(N/A^{2})$; R is the coil radius; I is the coil current; and l is the distance from the desired point to the center of the coil.

Thus, the relative position between the solenoid and the magnetic piston is constant, and the resistance and inductance of the solenoid are constant when the coil is wound uniformly. The attractive force $f_{G}$ of that electromagnetic structure is expressed as follows:

(11) $$f_{G}=K_{c}I,\;\mathrm{where}\;K_{c}=\frac{N^{2}\mu_{0}^{}S}{2K_{f}^{2}},$$

where N is the number of coil turns; S is the cross-sectional area of the magnetic circuit; and $K_{f}$ is the leakage coefficient, affected by the structure and material of the magnetic circuit.

## 4. Material Object Design

### 4.1. Electromagnetic Structure

**Figure 6:**
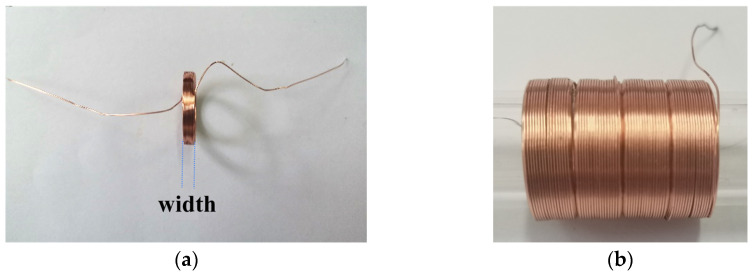
Physical layout of the solenoid: (**a**) Diameter of the copper wire used in the small coil is 0.3 mm, and the width is 3 mm. (**b**) Eight small coil connections.

Realizing the solenoid winding model designed can be challenging during the production process, primarily due to the small diameter of the copper wire, limited by the workmanship and formed by splicing a plurality of small coils of traditional winding. As shown in Figure 6a, the copper wire diameter used for the small coil is 0.3 mm, with a width of 3 mm. The welding of two small coil joints will inevitably cause gaps when splicing. The gap size is determined by the welding length reserved line and the welding point process. Figure 6b shows the local connection, revealing the connection of eight small solenoids. As shown in this figure, the gap between the solenoids becomes small when the connection point is on the inner side of the coil. The gap is large when the connection point is on the outer side of the coil, approximately twice the diameter of the copper wire. Table 1 shows the solenoid parameters.

**Figure 7:**
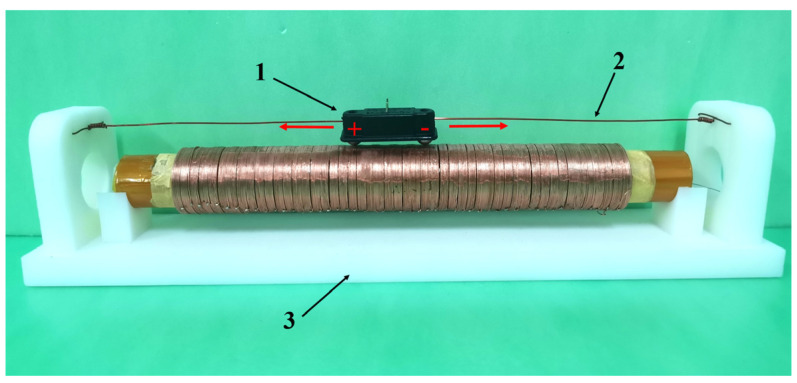
Solenoid power supply device: 1. Magnetic Globule Fixing Cap. 2. Trajectory Guide Line. 3. Support Structure.

An enameled copper wire is used for the solenoid. The insulating paint is scraped off at the outermost layer part to guarantee its proper functioning. This scenario allows two small magnetic globules to be used as the access points of the solenoid. These globules are fixed in the black cap-shaped structure to prevent any short circuit caused by the adhesion of the two small magnetic globules during movement. Moreover, the spin of the magnetic globules is restrained to ensure the attraction of the magnetic globules to the inner piston stability. Moreover, the track line passes through the fixing cap and is connected to the fixing plate to limit the motion track of the magnetic globules. In addition, magnetic globules are connected to the power supply through a cap-shaped structure to facilitate the best power supply connection and prevent disconnection. Figure 7 shows that the solenoid is energized.

### 4.2. Piston Structure

**Figure 8:**
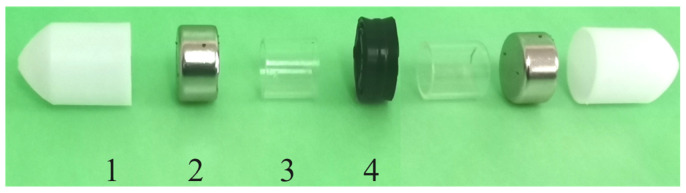
Magnetic piston structure. 1. Wrapping Shell. 2. Permanent Magnet. 3. Counterweight Support. 4. Rubber Ring.

The piston must divide the left and right gas chambers for optimal functioning. Moreover, the physical design must guarantee gas tightness to avoid gas backtracking caused by the pressure difference between the two sides of the piston. Additionally, minimizing the sliding friction between the piston and the inner wall of the circular tube is crucial to reduce energy loss. The piston structure consists of four parts: Wrapping Shell, Permanent Magnet, Counterweight Support, and Rubber Ring. Refer to Figure 8 for a clear visual representation of the piston structure.

The magnetic piston has an overall length of 55 mm and comprises two powerful cylindrical permanent magnets (2) with opposite magnetic poles at each end. The outer part of the magnets is wrapped by the wrapping shell (1) with an outer diameter of 16 mm. The wall thickness is 0.5 mm, and the overall length is 15 mm. Both ends are in bullet head shapes to reduce motion resistance. The diameter of the rubber ring (4) is 16 mm; the width is 8 mm to reduce friction and increase air tightness during movement. A small amount of lubricating liquid is coated on the outer surface of the rubber ring to reduce the friction force and increase air tightness during movement. However, the counterweight support (3) connects the magnets at the two ends and the middle rubber ring. The counterweight support is used for adjusting the total weight of the piston. The three parts are coaxially connected, and the piston has sealing and guiding functions. Table 2 shows the structural parameters of the piston.

### 4.3. Air Tightness and Friction Verification

**Figure 9:**
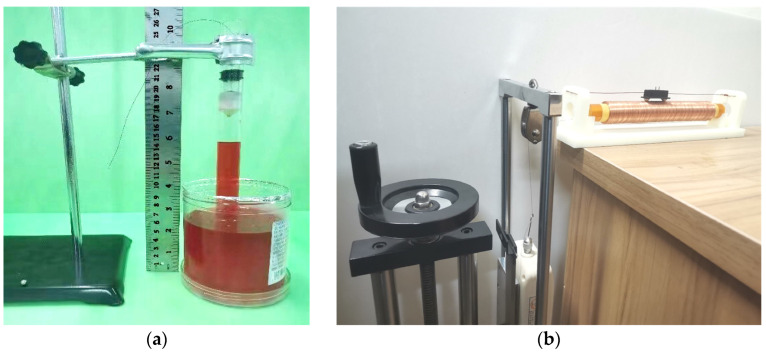
Verification of the airtightness and friction of the piston. (**a**) Piston tightness test. (**b**) Piston friction experiment.

Putting the round pipe vertically in the water tray is a restorative procedure during the air tightness test of the structure. The next step is to drag the internal piston upward and raise the water column to 10 cm, a position higher than the average water level under the action of atmospheric pressure. At this time, the water column should be allowed to stand for 1 min and the piston position should remain unchanged to observe the falling height of its liquid level, as shown in Figure 9a.

Our multi-group testing shows that the liquid level drops by approximately 0.4 cm after standing, which accounts for roughly 4% of the total height. In practical application, the air pressure at the two ends of the piston experiences oscillation, preventing long-time compression in a single direction. Additionally, the oscillating period is far less than 1 min, ensuring that the air tightness of the structure meets the application requirement.

Figure 9b shows the piston dynamic friction test. During measurement, the two ends of the circular tube are opened; the power supply is cut off, and the piston is subjected to the traction force $F_{tf}$ (tractive force) and the friction force $f_{f}$ of the dynamometer in the horizontal direction at the moment. In the entire motion process, the piston motion speed is zero at the starting and ending moments. As per the law of conservation of energy, the work done by the traction force is equal to that done by the friction force.

(12) $$\int_{0}^{s}f_{f}(s)ds=\int_{0}^{s}F_{tf}(s)ds,$$

where s is the relative position of the piston in the tube. Thus, (12) shows that when the traction force moves in a uniform straight line, it is equal to the friction force.

**Figure 10:**
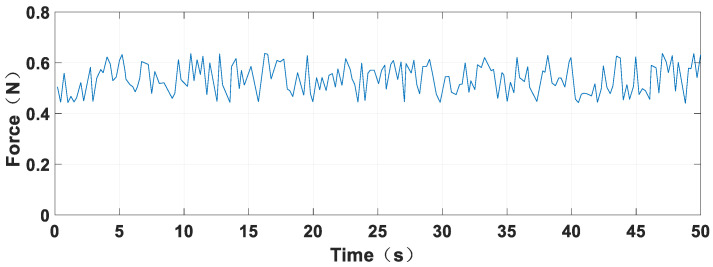
Friction measurement data.

Figure 10 shows the dataset in the friction force measurement process, indicating that the friction force generated in the measurement process fluctuates between 0.45 and 0.65 N. The fluctuation is due to the uneven movement process of the piston structure, leading to system acceleration. Moreover, the spliced solenoid outer surface generates an inevitable gap that further contributes to this fluctuation.

## 5. Parameter Analysis and Control Simulation

### 5.1. Analysis of Parameters

According to the mathematical model of the air supply structure, several main parameters impact the natural oscillation characteristics of the structure. These parameters are the piston mass, pipe length, cross-sectional area, the initial position of the piston and the initial pressure in the tube. Notably, the two initial values of the initial air pressure in the pipe and the initial position of the piston must be set according to the actual situation. First, changing the pipe’s initial air pressure will affect the structure’s pressure supply range and total output air pressure. These changes are determined according to the working air pressure range of the soft robot in practical applications. In this paper, the initial air pressure p in the tube is set as 10 kPa.

Second, the effect of changing the piston’s initial position on the structure’s inherent characteristics is similar in principle to that of changing the length of the circular tube at the same initial position [34]. At the starting time, the pre-input force of the electromagnetic structure is used to make the initial position of the piston $x_{0}\neq 0$. The piston performs a sinusoidal-like motion with amplitude attenuation in the structure due to the influence of friction force. The initial position of the piston is far from the center of the circular tube to make the vibration effect obvious. However, considering that it is difficult to provide excessive pre-input electromagnetic force using the actual equipment, this paper sets the initial position as $x_{0}=10\;cm$, making the simulation effect significant and the demand for pre-input electromagnetic force more reasonable.

Three variables are identified to simplify the structural model design: piston mass, tube length, and cross-sectional area. The inherent vibration frequency of the structure is negatively related to the piston mass and the pipe length, and positively related to the radius (cross-sectional area) of the tube [34]. Different parameter combinations can be selected for different working environments in practical applications. Conducting the correct analysis of each scenario requires selecting different parameter combinations. This article selects three parameters: combination C for high-load, high-stability operations; combination A for low-load, high-accuracy operations; and combination B as a compromise for general working conditions. As shown in Table 3, taking the oscillation characteristics when the friction force $f_{f}=0$ as the reference group, the self-oscillation simulation experiment is performed for each group of parameters, and the simulation results are analyzed.

**Figure 11:**
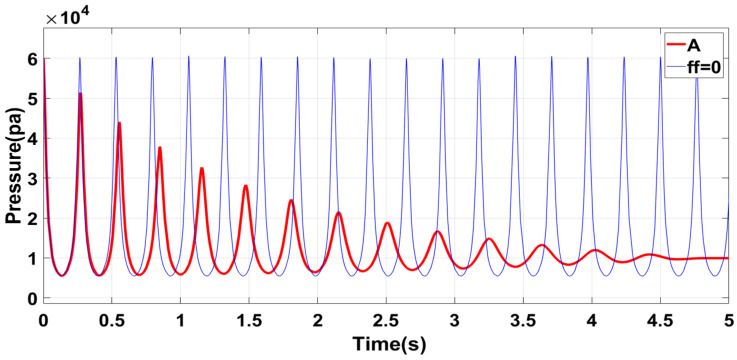
Group A structural characteristics.

Figure 11 shows that the oscillation frequency of the system is higher with a lower mass piston and a larger radius circular tube. This process increases the system’s flexibility, which can be advantageous in certain applications. However, the smaller mass of the piston reduces its corresponding inertial potential energy, making it easier to change the motion state. These changes can decrease the system’s stability, which must be considered when evaluating different applications. Group A is recommended for applications that demand equipment with high flexibility and accuracy but do not require high gas pressure output. The performance of Group A aligns with the expected effect and can be a suitable option for specific applications.

**Figure 12:**
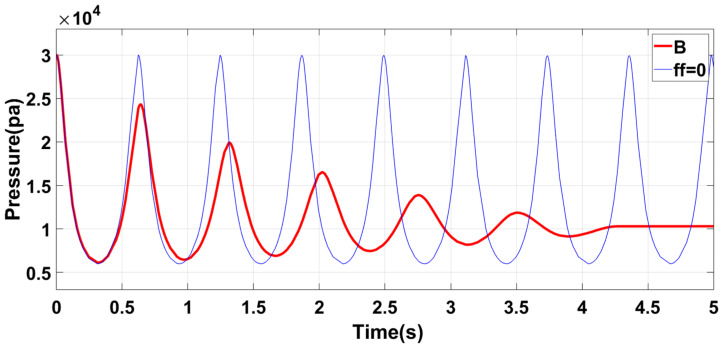
Group B structural characteristics.

Figure 12 shows that Group B has significantly lower air initial pressure than Group A. This is because increasing the length of the circular tube causes a reduction in the gas pressure at the left and right ends of the piston, decreasing the acceleration and speed of piston movement and increasing the self-oscillation period of the piston. To ensure the accuracy and flexibility of the system, Group B improves the upper limit of the output air pressure of the structure, improves the system’s stability, and enhances the wider practicability.

**Figure 13:**
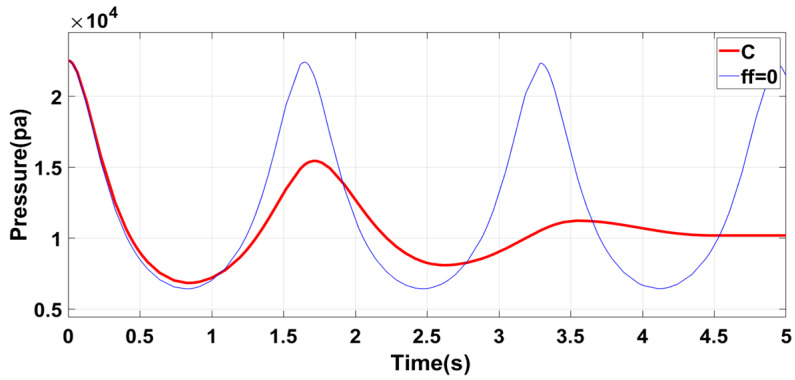
Group C structural characteristics.

Upon examining the Group C structural system, its increase in self-oscillation and reduction in the initial pressure positively impacted the system’s stability and output air pressure, as shown in Figure 13. The larger inertial potential energy of the piston makes it difficult to alter its state movement, ultimately increasing the system’s stability. Furthermore, the larger mass enabled the piston to move further toward high pressure with greater ease, raising the upper limit of the output air pressure of the structure. The downside to this process was that the larger length of the tube and piston’s mass greatly reduced the system’s oscillation frequency, weakening the flexibility and system accuracy. Consequently, the C group structural parameters are best suited for working environments with relatively stable movement, relatively smooth air pressure-demand, large output pressure, and low flexibility requirement.

### 5.2. Neural Network PID Control Strategy

The proposed piston structure exhibits the following key characteristics:

1. Nonlinearity of the system. First, a strong nonlinearity is noted between the input and output due to the friction force that reduces the amplitude of the piston during movement. Moreover, when the piston is close to an endpoint of the structure (such as the right endpoint), the gas pressure on the right side increases rapidly, thereby preventing the piston from moving to the right side.
2. Second, the corresponding pressure of the expected curve is relatively smooth, which reduces the control difficulty, essentially facilitating the practical applications of the body’s stability and ensuring less vibration, offering limited required output force, and ensuring a continuous trend with no evident step.
3. Intrinsic oscillation characteristics of the system. The controller’s output is affected by the position error, initial state of the desired curve and respective movement trends of the desired pressure value. For further information on these inherent oscillation characteristics of the system, refer to Table 4.

In Table 4, sp is the expected air pressure value; pv is the inherent air pressure change trend of the system at the set time; $c^{+}$ indicates that the required output of the controller should increase relative to the output value of the traditional PID control; c is basically unchanged, and $c^{-}$ indicates that it should be appropriately reduced. When the state motions of sp and pv are opposite, the state is the $c^{+}$ state. To make the output pressure of the structure align with the expected value, the inertial force in the original movement direction of the piston must be overcome, in addition to the thrust produced by the gas in the pipe. The controller must exhibit a larger output force than the result calculated by the error. When the two state motions are the same, the state is the $c^{-}$ state; the motion direction of the piston is consistent with the expected direction. Moreover, the control output must change the magnitude of the piston’s motion speed, and less output force is required compared with the other two states.

**Figure 14:**
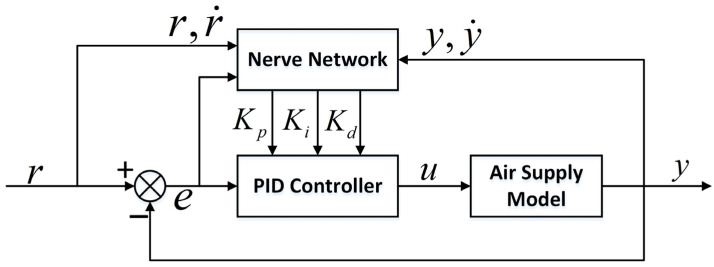
Neural network PID control block diagram.

Therefore, this paper introduces three parameters: the output of the control object, the first-order differential of the control object’s output, and the expected signal’s first-order differential based on the previous neural network PID controller. The overall control block diagram, considering the influence of the inherent vibration characteristics of the structure on the controller output, is depicted in Figure 14.

The output increment $\Delta u(n)=KX^{T}$ of the controller, where K is the parameter matrix to be optimized, and X is the neural network input matrix, given by

(13) $$\left\{\begin{array}{l} K=\left[k_{1},k_{2},k_{3},k_{4},k_{5},k_{6}^{}\right]\\ X={\left[\begin{array}{l} e(n)\\ e(n)-e(n-1)\\ e(n)-2e(n-1)+e(n-2)\\ y(n)\\ y(n)-y(n-1)\\ r(n)-r(n-1) \end{array}\right]}^{T} \end{array}\right..$$

We estimate that the pressure difference $\left|f_{r}-f_{l}\right|$ generated by the air pressure at the left end and right end of the piston accords with the maximum value of the expected air supply pressure. Moreover, the maximum value ${\left|f_{r}-f_{l}\right|}_{\max}$ of the pressure difference appears at the endpoint of a designed motion interval.

At time $t_{0}$, u(n−1)=0, let $\Delta u(n)={\left|f_{r}-f_{l}\right|}_{\max}$, and let that output at this point be supplied only by the gain term $k_{1}$; then, $k_{1}=\Delta u(n)/x_{0}$, and initialize $k_{3}=k_{2}=0.1k_{1}$, k4=k5=k6=0.

In the simulation model, the expected maximum air pressure is preset as $10^{5}pa$; then, ${\left|f_{r}-f_{l}\right|}_{\max}\approx 4N$, $k_{1}\approx 50$.

The output of the controller is given by

(14) u(n)=u(n−1)+Δu(n).

Assume that the general control object model is y=ψ(u), then

(15) y(n)=ψ(u(n)).

The objective training function is selected, taking the quadratic output error as the performance index:

(16) $$J=\frac{1}{2}{\left(r(n)-y(n)\right)}^{2}.$$

The gradient descent method is used to optimize the parameter K:

(17) $$\left\{\begin{array}{l} \Delta k_{i}=\eta e(n)\frac{\partial \psi (u(n))}{\partial u(n)}x_{i}\\ k(n+1)=k(n)+\Delta k_{i} \end{array}\right..$$

In this paper, $\frac{\partial \psi (u(n))}{\partial u(n)}$ is used instead of $\frac{\psi (u(n))-\psi (u(n-1))}{u(n)-u(n-1)}$ because the controlled object is challenging to obtain. Moreover, the error value is reduced by adjusting the value of η.

The method of changing the frequency, amplitude and phase of the sine wave from time to time is used as the data input, and the training dataset is formed by combining it with the feedback state of the system. Algorithm 1 shows the pseudocode of the control program.
**Algorithm 1. Air Pressure Control**Input: Expected air pressure curve p(t), initial parameters $k_{1}-k_{6}$ of the neural network Output: Control voltage u t←t+dt sp_p←p(t) read(pv_p) error_p←sp_p−pv_p error_sp←sp_p−sp_p_old error_pv←pv_p−pv_p_old $\Delta u\leftarrow k1*error\_p+k2*\left(error\_p-error\_p\_1\right)$  $+k3*\left(\;error\_p-2*\;error\_p\_1+error\_p\_2\right)$  +k4∗ pv_p+k5∗ error_pv+k6∗ error_sp control←control+Δu $k_{i}\leftarrow k_{i}+\eta *error\_p*\left(\;error\_pv/\Delta u\right)*x_{i},i=1\;to\;6$ sp_p_old←sp, pv_p_old← pv_p  error_p_2← error_p_1,  error_p_1←error_p


The core purpose of the neural network PID control method is to establish a PID controller that can realize the controller parameters’ self-tuning, enhance the automatic real-time adjustment of the controller parameters, and enable the gas supply structure to provide a continuous pressure supply curve within a certain amplitude range.

The control method used in this paper should be compared with other advanced nonlinear control methods, and has the following characteristics:

1. The controller based on input and feedback reduces the requirement of system accuracy.
2. Using the adaptability of the neural network, the best linear combination is found from the complex PID three parameters combination, and the automatic real-time adjustment of parameters is realized, which can effectively improve the robustness and reliability of the system.
3. The introduction of piston position and its motion state can produce good prediction ability for model parameters and controller output.

### 5.3. Air Pressure Control Simulation

**Figure 15:**
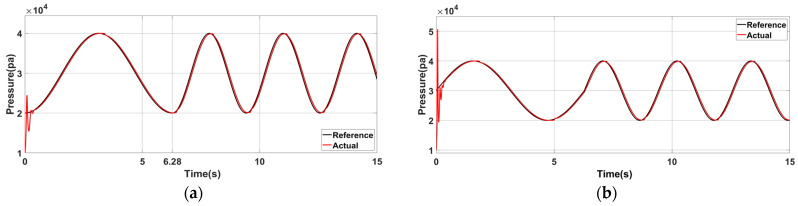
Simulation results of air pressure control when the model parameters are Group A: (**a**) The initial state is $\varphi_{0}=-0.5\pi ,\;\omega_{0}=1$; the desired frequency is changed to $2\omega_{0}$ at t=2π. (**b**) Air pressure control effect after the initial phase is changed to $\varphi_{1}=0$.

The control simulation experiments conducted on three groups of communication structures with different model parameters show that the expected pressure curve is relatively smooth. Table 5 presents the simulation model and expected curve parameters. Figure 15 shows the simulation results.

Under the proposed control strategy, the control simulation is conducted for the structure with the parameters of Group A, and the control result is shown as Act in Figure 15a. In the initial control stage, a large deviation is shown between the expected and inherent pressure oscillation curves. The tracking effect is stable when the controller is adjusted for a short time. No larger error is generated when the desired frequency is changed because the position and speed are continuous before and after the change in the desired air pressure, compared with the initial control stage.

Figure 15b shows the air pressure control curve at the initial phase $\varphi_{1}=0$ of the expected curve. Compared with Figure 15a, the actual output curve at the initial stage increases because the initial state of (a) is close to the initial state of the equipment’s inherent oscillation characteristics, making switching between the two relatively smooth. The results highlighted the importance of choosing the appropriate adjustment time node, which can significantly reduce energy consumption and ensure stability.

**Figure 16:**
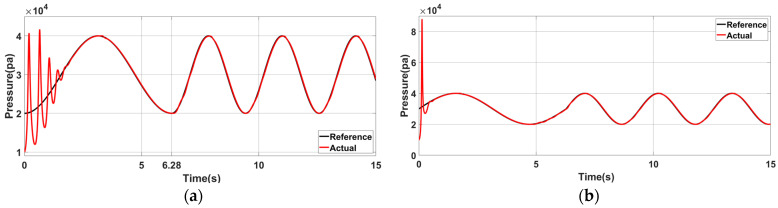
Simulation results of air pressure control when the model parameters are Group B: (**a**) The initial state is $\varphi_{0}=-0.5\pi ,\;\omega_{0}=1$; the desired frequency is changed to $2\omega_{0}$ at t=2π. (**b**) Air pressure control effect after the initial phase is changed to $\varphi_{1}=0$.

Figure 16 shows that Group B structural parameters significantly impact control simulation results. Compared with Figure 15, the initial air pressure control error is higher, and the adjustment time is also prolonged due to the increased length of the tube and mass of the piston, resulting in higher inertial potential energy of the piston. Consequently, its initial and motion states become more challenging to alter, leading to a longer adjustment time of the initial control.

**Figure 17:**
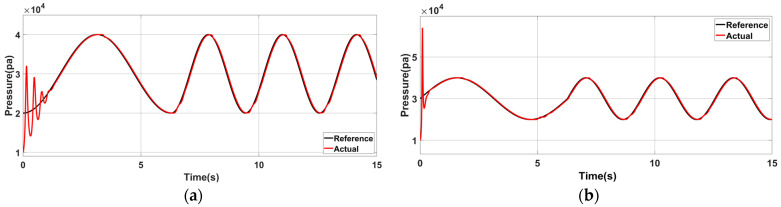
Simulation results of air pressure control when the model parameters are Group C: (**a**) The initial state is $\varphi_{0}=-0.5\pi ,\;\omega_{0}=1$, and the desired frequency is changed to $2\omega_{0}$ at t=2π. (**b**) Air pressure control effect after the initial phase is changed to $\varphi_{1}=0$.

Figure 17 shows that the control error, adjustment time and initial controller output increase significantly compared to those in Figure 15 and Figure 16. The pressure control curve for each group highlights the maximum deviation of air pressure control that occurs at the initial time. There is a significant difference between the initial value of the initially expected pressure curve and the initial value of the inherent pressure oscillation curve, requiring a brief process for adjusting the controller parameters. The controller can adapt well to the process as the system tends to be stable, and the frequency of the desired curve changes again, producing a slight fluctuation in the control error. It is also observed that the simulation error would fluctuate slightly due to the change in motion direction at the stable moment. In the actual moving process, the proposed structure can fully use the energy stored after gas compression to push the piston and reduce the external energy input needed in the subsequent movement. Therefore, the proposed structure can fully use the compressed gas energy stored to reduce the system’s overall energy consumption.

## 6. Physical Verification

### 6.1. Air Pressure Control Experiment Platform

**Figure 18:**
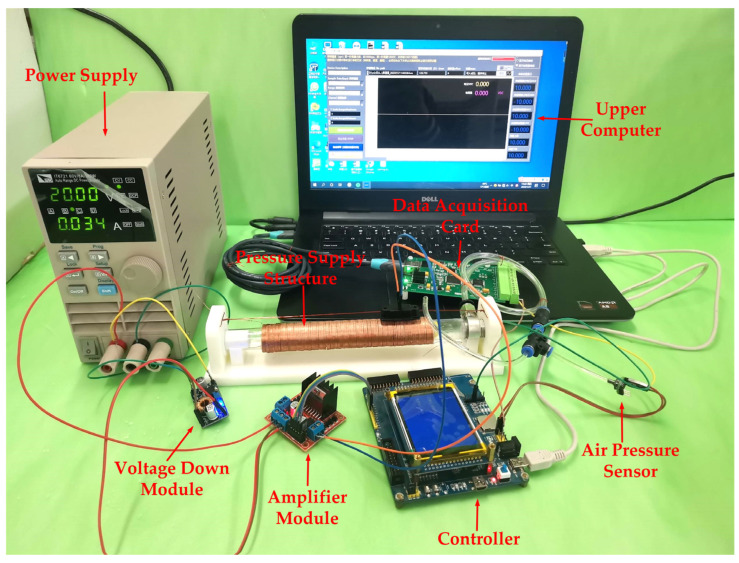
Experimental platform for pressure control based on XGZP6847D pressure sensor.

An experimental platform has been designed to verify the air pressure supply characteristics of the structure. The platform uses an experimental air pressure control platform based on the XGZP6847D air pressure sensor developed by CFSensor, as shown in Figure 18. The sensor power supply voltage is 3.3 V. The output voltage is 0.2–2.7 V; the corresponding air pressure value is 0–200 kPa. The experimental data are displayed in real-time on the host computer interface through the 16-bit data acquisition card.

The left end of the air supply structure is simplified through the experimental process, connecting the right end of the internal pressure to the three-way valve, screw cap, and air guide hose. Moreover, the valve is connected to the interface of the pressure sensor to measure the internal pressure. A computer USB independently powers the low-voltage control circuit, measurement circuit, and acquisition circuit to facilitate the maximum voltage value fed back by the air pressure sensor. This process is to ascertain that the system does not exceed the maximum range of the A/D conversion module. Moreover, the data acquisition card transmits the acquired data to an upper computer in real-time through a USB interface, allowing the air pressure to conveniently change the movement.

### 6.2. Air Pressure Tracking Effect Experiment

**Figure 19:**
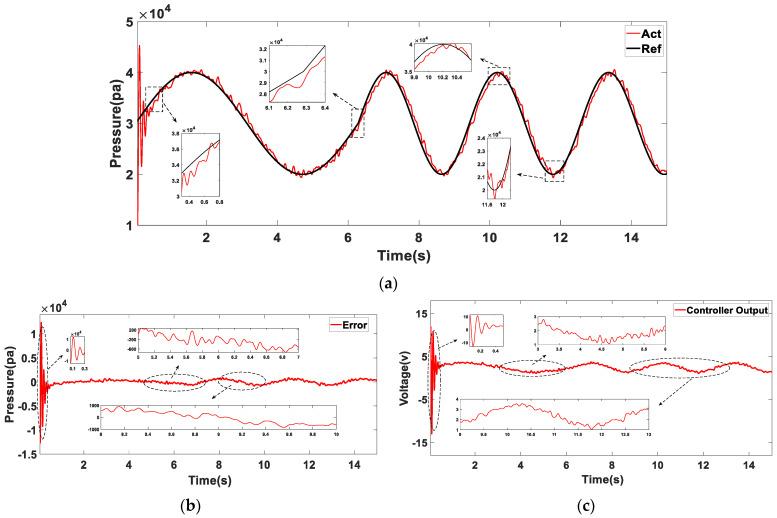
The initial state is $\varphi_{0}=-0.5\pi ,\;\omega_{0}=1$; the desired frequency is changed to $2\omega_{0}$ when t=2π. The experimental results were analyzed. (**a**) Air pressure tracking effect; (**b**) Control error; (**c**) Controller output.

The controller’s initial parameters in the prototype experiment use the simulation training’s final results. In the physical verification, the learning rate is appropriately reduced to avoid the disproportionate impact of noise interference on the control parameters. The tracking characteristics of sinusoidal pressure are analyzed. In the zero states, the initial state of the desired air pressure is set to $\varphi_{0}=-0.5\pi ,\omega_{0}=1$. In the t=2π state, the desired frequency is changed to $2\omega_{0}$. In the experiment, observing the actual pressure curve change rule is challenging because the pressure feedback value has many sampling points. Moreover, the signal fluctuates frequently. Then, simple median filtering is conducted after exporting the collected pressure data. Figure 19 depicts the experimental results.

In the initial control stage (0~0.3s), as shown in Figure 19b, the fluctuation range of the control error is ±12kPa. The error is large, consistent with the simulation result. From the output curve of the controller, as shown in Figure 19c, when the desired air pressure frequency changes because the position and speed are continuous before and after the change, the controller’s output decreases compared with the initial control stage. After reaching the desired frequency, the controller’s output only compensates for the energy system consumption.

When the control signal is gradually stabilized, the control voltage is maintained between 1 and 4 V and changes relatively smoothly, as shown in Figure 19c. From the fluctuation of air pressure change after stabilization, as shown in Figure 19b, the error range is ±0.8 kPa when the air pressure is in the peak or trough. Compared with the error range of ±0.3 kPa in the rising or falling stage, the jitter is larger. On the one hand, because the piston is in an endpoint in the movement process, the movement direction will change, and the structure will produce a certain vibration. On the other hand, because the friction direction changes at the moment, the controller’s output is unstable, resulting in relatively frequent changes in internal air pressure.

The overall trend of the actual pressure curve shows a high degree of similarity with the simulation results. Smoothly adjusting the preset air pressure change frequency during movement does not have a significant effect on the controller output. The experiment shows that the proposed air supply structure and improved control algorithm have a better tracking effect on the changes in sine pressure.

### 6.3. Practical Analysis

The typical pneumatic soft structures are PAMs, soft rehabilitation robots, and flexible manipulators. The working air pressure range of the flexible bionic mechanical arm is between 60 and 250 kPa [35,36,37]. The maximum working pressure of the soft hand can reach 70 kPa [38,39]. The working pressure range of the soft quadruped robot [40] is between 150 and 218 kPa. The working pressure of the flexible bionic robot fish [24] reaches 80 kPa. Refer to Table 6 for application examples of various typical pneumatic soft robots and their corresponding working pressure ranges.

The air supply structure designed can produce a continuous pressure supply curve, fluctuating in the range of 10–80 kPa, to meet the pressure supply requirements of most of the soft structures. As for the soft robot exceeding the air pressure range supplied by the structure, an air pressure supply curve that fluctuates in a larger range can be generated by increasing the pipe’s initial air pressure and control current to meet the pressure supply requirement. Therefore, the research shows that the structure can be widely used in the air pressure supply of soft robots.

## 7. Conclusions

This study proposes a new pneumatic supply structure to solve the problems of large volume, low stability, and high energy consumption in pneumatic soft robots; according to the working principle, dynamic characteristics, manufacturing method, control method and practicability of the structure. The main innovations of this paper are as follows:

1. The pressure supply structure proposed in this paper has the characteristics of light weight, small volume, easy assembly and so on. Experiments show that the pressure supply structure can meet the demand of pressure supply, and can replace the traditional gas supply mode in most occasions, which solves the problem of carrying common gas supply equipment, provides a new scheme for the research of miniaturized pressure supply device, and has a wide application prospect in the application field of outdoor soft robot.
2. The pressure supply structure proposed in this paper can effectively use the energy stored in the compressed gas and reduce the overall energy consumption of the system. The inherent oscillation characteristics of the structure enable the energy stored by the compressed gas inside to be used to push the piston to move reversely, thus reducing the energy input of the subsequent process. It solves the problem of low energy utilization of traditional gas supply equipment and provides a new solution for the research on low energy consumption pressure supply devices.
3. The characteristics of the model are analyzed in detail, and the main parameters that affect the characteristics are estimated and compensated for, a neural network control algorithm is improved, and the self-tuning process of the controller parameters under different conditions is realized, which provides a new design idea for the control strategy of such nonlinear systems and has reference significance.

The winding mode of the electromagnetic structure used in this paper is a new type of winding mode, and the feasibility of the theoretical model of the pressure supply structure is verified by the way of experimental research on the specific object. However, further research on the relationship between the main parameters of the electromagnetic structure model and electromagnetic force may have more gains and discoveries. At the same time, the influence of different loads on the gas supply model remains to be further studied.

## Figures and Tables

**Table 1:** Physical parameters of the solenoid.

Dimensions (Units)	Copper Conductor	Small Coil	Solenoid	Lateral Space
Diameter (mm)	0.3	-	-	-
Inside Diameter (mm)	-	18	18	-
Number of Turns (n)	-	20	20	-
Number of Cycles (n)	-	10	-	-
Width/Length (mm)	-	3	240	0.6

**Table 2:** Physical parameters of the piston.

Dimensions (Units)	Magnet	Wrapping Shell	Rubber Ring	Piston General
Length (mm)	8	15	10	55
Diameter (mm)	15	-	16	16
Outside Diameter (mm)	-	16	-	-
Thickness (mm)	-	0.5	-	-
Number (n)	2	2	1	1

**Table 3:** Air supply structure self-oscillation simulation parameter grouping.

Group	Piston Mass (g)	Tube Length (cm)	Cross-Sectional Area (cm^2^)
A	100	12	0.48π
B	200	15	0.32π
C	500	18	0.16π

**Table 4:** Classification of control state.

Trend of Change	*sp* Increase	*sp* Maintain	*sp* Decrease
pv Increase	$c^{-}$	c	$c^{+}$
pv Maintain	c	c	c
pv Decrease	$c^{+}$	c	$c^{-}$

**Table 5:** Model structure and input signal parameters.

Parameter	Value (Units)	Input Signal	Value (Units)
Number of Coil Turns (N)	20	$\varphi_{0}$	−0.5π
Magnetic Cross-Section (S)	$s\;({\mathrm{cm}}^{2})$	$\varphi_{1}$	0
Resistance (R)	40 (Ω)	$\omega_{0}$	1
Magnetic Leakage Coefficient ($K_{f}$)	2	t	2π (s)

**Table 6:** Research on typical pneumatic software structure and analysis of working air pressure range.

Correlative Research	Pneumatic Soft Structures	Operating Pressure Range (kPa)
McKibben et al. [27] Guan et al. [28] Rodrigue et al. [34]	Pneumatic Artificial Muscle	−100 to 800
Ge et al. [29]	Soft Rehabilitation Gloves	20–30
Marchese et al. [36] Mahl et al. [37] McMahan et al. [38]	Flexible Mechanical Arm	60–250
Martinez et al. [39] Hao et al. [40]	Soft Hand Grasp	30–70
Tolley et al. [24]	Soft Quadruped Robot	150–220
Katzschmann et al. [41]	Soft-Bodied Mechanical Fish	40–80

## Data Availability

Data is contained within the article.
